# Opposing Timing Constraints Severely Limit the Use of Pupillometry to Investigate Visual Statistical Learning

**DOI:** 10.3389/fpsyg.2019.01792

**Published:** 2019-08-06

**Authors:** Felicia Zhang, Lauren L. Emberson

**Affiliations:** Department of Psychology, Princeton University, Princeton, NJ, United States

**Keywords:** pupillometry, learning, prediction, pupil dilation, visual statistical learning

## Abstract

Majority of visual statistical learning (VSL) research uses only offline measures, collected after the familiarization phase (i.e., learning) has occurred. Offline measures have revealed a lot about the extent of statistical learning (SL) but less is known about the learning mechanisms that support VSL. Studies have shown that prediction can be a potential learning mechanism for VSL, but it is difficult to examine the role of prediction in VSL using offline measures alone. Pupil diameter is a promising online measure to index prediction in VSL because it can be collected during learning, requires no overt action or task and can be used in a wide-range of populations (e.g., infants and adults). Furthermore, pupil diameter has already been used to investigate processes that are part of prediction such as prediction error and updating. While the properties of pupil diameter have the potentially to powerfully expand studies in VSL, through a series of three experiments, we find that the two are not compatible with each other. Our results revealed that pupil diameter, used to index prediction, is not related to offline measures of learning. We also found that pupil differences that appear to be a result of prediction, are actually a result of where we chose to baseline instead. Ultimately, we conclude that the fast-paced nature of VSL paradigms make it incompatible with the slow nature of pupil change. Therefore, our findings suggest pupillometry should not be used to investigate learning mechanisms in fast-paced VSL tasks.

## Introduction

Statistical learning (SL) is the ability to exploit patterns in the environment after passive exposure. SL has been demonstrated using a wide range of stimuli (e.g., Aslin, 2017; Saffran and Kirkham, 2018) and is considered a domain general learning mechanism used by cognitive systems to detect structure in the environment. Since SL is available starting early in life, it is believed to be important for development (Krogh et al., 2013; Armstrong et al., 2017). This paper focuses on visual statistical learning (VSL), or SL paradigms using visual stimuli and investigates whether pupillometry (the measurement of pupil diameter), could be effectively used to investigate the mechanisms supporting VSL.

A standard VSL paradigm is composed of two phases: Familiarization and test. During familiarization, participants are shown a stream of images one at a time, for ∼1000 ms, without explicit instructions. The images are organized based on statistical information (e.g., transitional probability, co-occurrence frequency) such that some of the images are more likely to appear one after another compared to others. For example, a stream of images can be composed of triplets, such that the same three images always appeared in the same sequence (ABC-DEF-ABC-GHI) and statistical information is the only cue that learners have to uncover the structure or organization of the visual stream. The second phase is test phase, where participants’ learning of the statistical information is probed by forced-choice judgments (e.g., A vs. B) (Turk-Browne et al., 2005; Brady and Oliva, 2008; Turk-Browne and Scholl, 2009) or familiarity ratings for single items (Baker et al., 2004; Turk-Browne et al., 2010b). Following common nomenclature in the field, we will refer to these measures as **offline measures** because they are collected after learning (i.e., after the familiarization phase or during the test phase) has occurred. Conversely, we will refer to measures collected *during* learning (i.e., during the familiarization phase) as **online measures**.

Majority of the SL research conducted used only offline measures, and these studies have revealed a lot about the extent of SL, but are best suited for investigating what participants have learned rather than how they have learned. For example, VSL can operate over a variety of visual features (e.g., color and shape) and can even bind these features and objects (Turk-Browne et al., 2008). Furthermore, offline measures such as looking time have revealed that newborns (i.e., 1–3 day-old infants) are also able to learn simple visual statistical information (Bulf et al., 2011).

Offline measures, however, have their limitations (for a review see Siegelman et al., 2017). One of the biggest limitations, which is the focus of this paper, is that it is very difficult to use offline measures to investigate the online learning mechanisms that support SL. Important questions such as “what mechanisms allow for such rapid learning of this statistical information?” and “how do mechanisms that support SL change throughout development?” are still points of active investigation, in part, because of the reliance on offline measures.

While it is likely that multiple cognitive processes such as attention, memory and prediction interact online to enable VSL, this paper focuses on how **prediction**, or the ability to use past experiences to generate expectations about future sensory input, supports SL. This work unifies theories that have proposed that prediction is a core aspect of adult brain function (Rao and Ballard, 1995; Friston, 2005; Clark, 2013) and SL studies that suggest prediction is at play (Turk-Browne et al., 2005; Kim et al., 2009; Siegelman et al., 2018). However, it is very difficult to investigate the role of prediction in VSL using offline measures alone.

The shortcomings of offline measures can be overcome by including online measures into SL paradigms. Online measures lend themselves to investigating how representations are formed during learning. For example, if a measure is able to reflect a process like prediction, using such measures can provide a window into how prediction operates during SL. Furthermore, online measures are helpful for revealing the trajectory of learning, both between trials and on a trial-by-trial basis within an individual, without repeated testing.

Recent SL studies have started using online measures. Electroencephalography (EEG) is a common tool used to record online measures of SL (Abla et al., 2008; Abla and Okanoya, 2009; Batterink and Paller, 2017). Event-related potentials (ERPs) such as the N400 have been shown to be different for good versus poor learners (Abla et al., 2008; Abla and Okanoya, 2009). Furthermore, reaction time (RT) is the most common behavioral online (Baker et al., 2004; Gómez et al., 2011; Franco et al., 2015).

Online measures can also be combined with offline measures in a single SL task. This combination is a powerful approach to examining the underlying cognitive processes that support learning and how that intertwines with what people learn. Indeed, Siegelman et al. (2018) found that the online measure of RT predicted offline measures of learning: participants who had faster RTs for predicted stimuli also performed better in the test phase (for similar results using a self-paced artificial grammar learning task see Karuza et al., 2014b).

Despite the usefulness and broad use of RT as an online measure of SL, it has substantial drawbacks. First, RT is most commonly gathered through button pressing, and this type of task is only possible for some populations. For example, button pressing cannot be used to investigate SL of infants and is very difficult for young children. Second, it has been argued that pressing buttons during familiarization interferes with learning (Franco et al., 2015). Relatedly, Turk-Browne et al. (2005) found that divided attention due to concurrent tasks can lead to poor learning as measured in offline tests. Finally, it has been suggested that RT measures are not stable. For example, Franco et al. (2015) found a different pattern of RT evolution in their click detection task compared to Gómez et al. (2011).

Building from these limitations of RT, an ideal online measure is one that reflects learning but requires no overt action or task and can be used in a wide-range of populations, ideally under identical experimental conditions. To this end, we propose **pupillometry**, or the measurement of pupil diameter, as an online measure for investigating SL. Pupillometry has none of the disadvantages of button pressing listed above and several additional advantages. First, pupillometry does not interfere with the learning process because it does not involve a secondary task. Pupillometry is automatically recorded from an eye tracker at extremely high temporal resolution. Second, due to the high temporal resolution, we record multiple measures during the same trial unlike RT, which is a summary measure. Sampling online processes throughout the presentation of a single stimulus can help to differentiate online learning mechanisms (e.g., processes that occur during stimulus processing versus in the anticipatory periods before a stimulus). Lastly and most importantly, changes in pupil diameter are time-locked to events and reflect processes that are part of prediction such as prediction error (i.e., incorrect predictions, Nassar et al., 2012; Koenig et al., 2018; Zhang et al., 2018) and updating of predictions (O’Reilly et al., 2013). Since pupillometry has been found to be sensitive to predictions, it may be able to evaluate the hypothesis that prediction is part of the learning mechanisms supporting SL.

**FIGURE 1:**
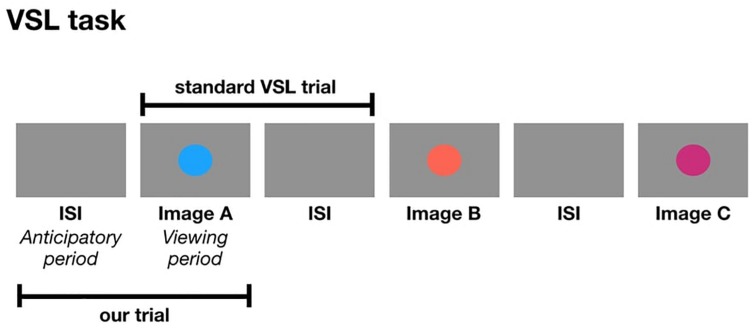
The formation of a trial in a standard VSL study compared to a trial in the current study.

The following studies are the first set of experiments to use pupillometry as an online measure during a VSL task. In a standard VSL task, a trial is composed of an image followed by the ISI. In the current study, a trial is composed of the ISI followed by the image. In other words, we are taking the ISI from the previous shape and considering it as the anticipatory period for the current image which is presented during the viewing period of a trial (Figure 1). One might ask: how do we know the pupil diameter during the anticipatory period is reflective of anticipation and not processing the visual stimulus from the previous trial? We can simply observe the pupil change to make that distinction. If the anticipatory period reflects lower-level visual processing, we would expect to see identical pupil change for all images. But if pupil change reflects prediction, then we expect to find pupil differences relative to image position in the triplet, and this difference may occur either in the anticipatory period or viewing period.

To compare pupil diameter within and across participants, we calculated pupil change from baseline for all experiments. We designated baseline as the anticipatory period of the first image in a structured sequence (i.e., triplets and pairs). This is a standard approach for pupillometry data (Preuschoff et al., 2011; Lavín et al., 2014; Kloosterman et al., 2015; Eckstein et al., 2016) and accommodates variations in pupil diameter due to individual differences and the dynamic changes that occur over the experiment.

If pupillometry reflects online mechanisms supporting VSL, we hypothesized that several factors that would influence pupil diameter:

1. **Image position within the triplet:** Based on previous studies demonstrating faster RT for predictable stimulus compared to unpredictable stimuli, and research suggesting that larger pupil diameter reflects prediction error, we hypothesize that the pupil diameter will differ for each image in a triplet during the viewing period. Specifically, we hypothesized that the pupil diameter will be the largest for the first image in the triplet (i.e., the least prediction image/largest prediction error) and smallest for the third image in the triplet (i.e., the most predictable) and the second image to fall in between. In our previous work with pupil size and passive learning in infants and adults, we find greater pupil size during violation trials (similar to unpredictable) compared to standard or predictable trials (Zhang et al., 2018). It is also possible that image position will influence pupil change during the anticipatory period, which would suggest that image position plays a role in temporal prediction (i.e., when will the visual stimulus appear). However, based on our previous work and other pupillometry work, we hypothesized the effect would show up during the viewing period if participants were predicting which image will appear instead of when.
2. **Number of times seen (i.e., familiarity effect**): During the familiarization phase of SL, participants are exposed to the stimuli multiple times. Research has shown that pupil dilation to be larger for familiar stimuli compared to novel stimuli (Kafkas and Montaldi, 2012, 2015). Therefore, we hypothesize that the more times you view an image, the larger the pupil change during the viewing period.
3. **Relationship to offline measures** (e.g., test accuracy): If pupil diameter is a suitable online measure for SL, it should be diagnostic of participants’ performance during test phase (i.e., correlated with offline measures). Kafkas and Montaldi (2011) had participants complete a “remember/know” task to examine the relationship between pupil change and memory strength. Participants incidentally encoded a series of images and were later asked to rate recognized items along a “strength” scale and to call unrecognized items “new.” The authors found that pupil constriction at encoding was associated with subsequently stronger memories. With respect to our study, we also expect to find pupil constriction to be related to better accuracy during test phase.

To foreshadow our results, while the combination of pupillometry and VSL has the potential to expand the online measures available to investigate VSL, we propose that the timing necessary to produce VSL is too fast to see the pupil effects that we hypothesized. In a series of three VSL studies, we found that pupil differences between image positions within a triplet to be a result of where we chose to baseline instead of online processes like prediction. Furthermore, we did not find pupil diameter to be related with offline measures of learning. We conclude the paper by discussing why pupillometry is not readily compatible with fast-paced VSL tasks and suggest alternative measures to use to examine learning during SL.

## General Methods

### Participants

One hundred and sixty-eight adult subjects (54 in Experiment 1; 61 in Experiment 2; 53 in Experiment 3), all with normal or correct-to-normal acuity and color vision, participated for course credit or monetary compensation ($6). Five subjects in Experiment 1 were excluded due to corrupted file (1 subject) and poor eye tracking (4 subjects). Eight subjects in Experiment 2 were excluded due to corrupted files (4 subjects) and poor eye tracking (4 subjects), which was defined as missing more than 25% of their data. Thirteen subjects in Experiment 3 were excluded due to poor eye tracking (same definition as above). Thus, data analysis in Experiment 1 included 49 participants, in Experiment 2 included 53 participants and in Experiment 3 included 40 participants. Experiments were approved by the University’s Institutional Review Board, and informed consent was obtained before beginning the study.

### Stimuli

Stimuli consisted of 12 fractal images (from Karuza et al., 2017, Figure 2). To prevent luminance-related changes in pupil diameter, all visual stimuli were made isoluminant (equal luminance) to the background color of the experiment and to the experimental room. The original fractal images were in black and white. To make the images isoluminant, we first had to add color to it. We used Photoshop CS6 (Adobe Systems, San Jose, CA, United States), imported the original images, created a new layer for each image and filled it with an orange color (#F28C09), set the layer blending mode to difference and exported the new colorful images. Next, we used an isoluminance script provided by Daniel Swingley (David Brainard and Daniel Swingley, personal communication, November 14, 2016) which takes a standard RGB image, corrects the gamma, separates the image’s chromaticity and luminosity, and sets the luminance to a constant value, thereby making the image isoluminant, without affecting the chromaticity. We selected a luminance value of 0.3 to match the lighting in the room and not distort the visual stimuli to the point of being unrecognizable.

**FIGURE 2:**
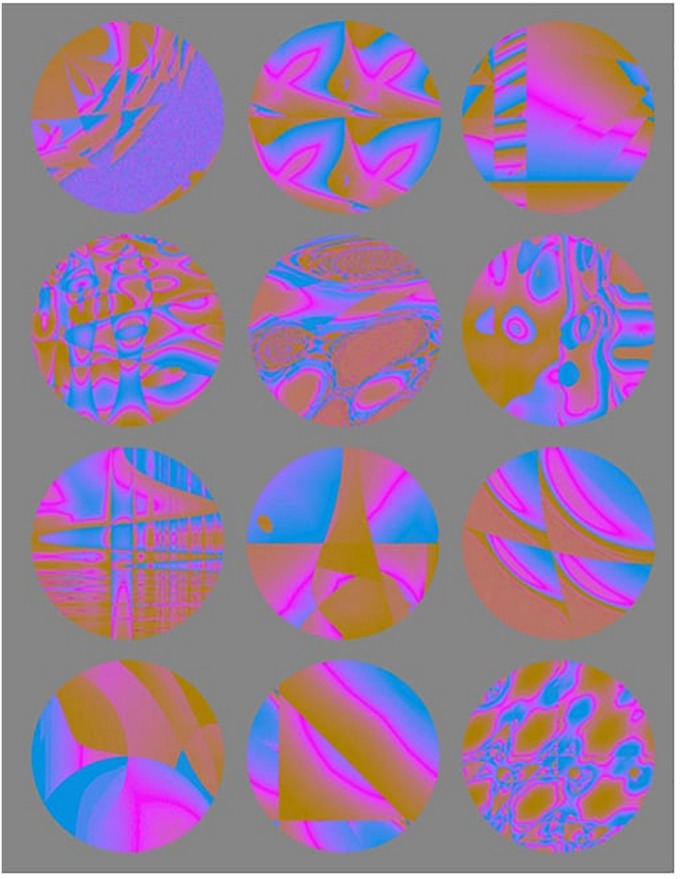
The 12 fractal images used in Experiment 1, Experiment 2 and Experiment 3.

### Apparatus

Participants sat approximately 60 cm from the monitor and eye tracker (Eyelink 1000, SR Research, Ottawa, ON, Canada). We set the eye-to-track parameter to “right.” The eye tracker records pupil diameter in arbitrary units at 500 Hz. Luminance in the room was measured using Light Meter, a mobile app used to measure light intensity (version 1.1, Elena Polyanskaya). The display monitor measured 34 cm by 27 cm and was facing the participant. The host monitor plus experimenting computer were in front of the experimenter. Before beginning the experiment, a five-point calibration was used. We performed calibration and validation for all participants.

### Procedure

The basis for the current experiments is the standard VSL paradigm. In Experiment 1, participants viewed a stream of visual stimuli one at a time with no explicit instructions during the familiarization phase. Unbeknownst to the participants, the stream of visual stimuli was composed of four possible triplets. In other words, the three images that made up a triplet, always appeared one after another (e.g., ABC-DEF-GHI-ABC). During the test phase, participants were explicitly tested on how well they learned the four triplets using a two-alternative forced-choice (2AFC) test. In Experiment 2, participants viewed a stream of visual stimuli during familiarization composed of paired images and unpaired images (e.g., AB-X-CD-Y-EF-Z-AB-Y) and were given a simple recognition test during test phase. In Experiment 3, participants viewed a stream of visual stimuli composed of triplets identical to Experiment 1, followed by a random block composed of the same images but presented in a randomized order. There was no test phase in Experiment 3. For all three studies, we defined the period when there is a blank screen (i.e., before the image appears) as the anticipatory period and the period when the image is shown as the viewing period. The anticipatory period was 500 ms and the viewing period was 750 ms, for a total trial duration of 1250 ms.

### Preprocessing

Data analysis only included participants with good validation (i.e., the highest validation accuracy). Missing pupil size samples, identified as blinks, were linearly interpolated. To compare pupil diameter within and across participants, we calculated pupil change from baseline for both experiments. Using pupil change from baseline instead of raw pupil measurement accommodates variations in pupil diameter due to individual differences and is a standard approach for pupillometry data (Preuschoff et al., 2011; Lavín et al., 2014; Mill et al., 2016). We defined baseline as the average pupil diameter during the anticipatory period of the first image in a group of three images. Next, the baseline was subtracted from each pupil diameter measurement in the group of three images and divided by the baseline to get the change from baseline. Then, we multiplied the number by 100 to convert the decimal number to a percentage. We will use the term **pupil diameter** to refer to the original pupil diameter recorded in arbitrary unit, **pupil change** to refer to the change in pupil size from baseline. To characterize types of pupil change, **pupil constriction** refers to decrease in pupil change from baseline and **pupil dilation** refers to increase in pupil change from baseline.

## Experiment 1: SL With Triplets and Two-Alternative Forced-Choice Test

**FIGURE 3:**
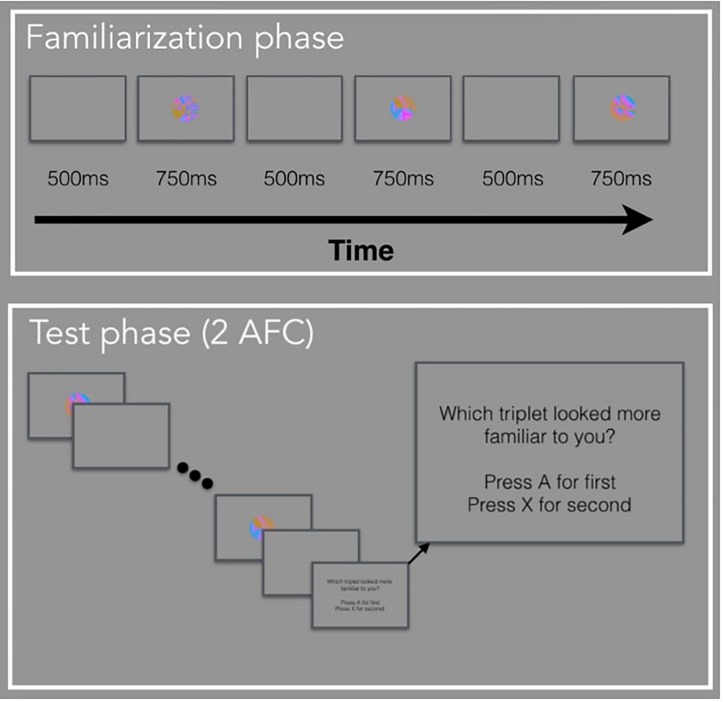
Schematic of VSL task in Experiment 1.

In Experiment 1, we measured adults’ pupil diameter as they participated in a typical VSL task. We modeled the paradigm after Turk-Browne et al. (2008), such that we included a passive exposure phase (i.e., familiarization phase) plus a surprise 2AFC test (i.e., test phase, Figure 3). This paradigm is suitable for analyzing individual differences because we can collect individuals’ pupil diameter during familiarization and learning outcomes. To analyze pupil diameter, we set the anticipatory period of the first image in a triplet or pair as baseline and calculated pupil change from baseline. We hypothesized that the pupil diameter will be different for each image in a triplet during the viewing period such that the pupil diameter will be the largest for the first image in the triplet (i.e., the least prediction image/largest prediction error) and smallest for the third image in the triplet (i.e., the most predictable) and the second image to fall in between. Furthermore, sensitivity to the order of the triplet images measured via pupil change will be related to performance during the test phase. We also calculated pupil change from baseline by using the anticipatory period of each image instead of the triplet. In Experiment 1 and subsequent experiments, we show that different selections of baseline can drastically alter results and lead to differences in pupil change unrelated to prediction.

In addition, we complemented these analyses by computing Siegelman et al. (2018)’s online measure of SL:

$$\begin{array}{c} \mathrm{average pupil change}\;(1^{\mathrm{st}}\;\mathrm{position})\\ \;-\mathrm{average pupil change}\;(2^{\mathrm{nd}}\;\mathrm{and}\;3^{\mathrm{rd}}\;\mathrm{position}) \end{array}$$

Siegelman et al. (2018) conducted a self-paced VSL paradigm, where participants were exposed to a stream of images composed of triplets and asked to press a button to view the next image. Using this formula with log-transformed RTs, Siegelman et al. (2018) found that participants learned the triplet structure after only a small number of exposures. Furthermore, the RT difference for a predictable vs. unpredictable image was highly correlated with learning outcome measured during test. We computed Siegelman et al. (2018) online measure of SL using pupil change and analyzed its relationship to performance on the test phase. Lastly, it is important to mention that given this novel combination of using pupillometry during a VSL task, our overall statistical plan is to engage in a series of exploratory analyses in Experiment 1, uncorrected, and then attempted to replicate and extend them in independent datasets (Experiments 2, 3).

### Procedure

#### Familiarization Phase

Participants watched a sequence of 288 images, presented in the center of the computer screen, one at a time. Unbeknownst to the participants, the sequence was composed of triplets, or sequences of three images that always appeared in the same order. We randomly assigned the 12 fractal images into 4 triplets (ABC, DEF, GHI, JKL) for each subject. The full sequence was generated by randomly interleaving eight repetitions of each triplet with two constraints (for a total of 24 repetitions of each triplet): (a) no triplet could be repeated sequentially (e.g., ABC-ABC), and (b) no pair of triplets could be immediately repeated (e.g., ABC-GHI-ABC-GHI).

#### Test Phase

Participants completed 32 two-interval forced-choice test trials, judging the relative familiarity of (a) triplets versus (b) foil sequences of three familiar images that had never appeared sequentially during familiarization. The foil triplets were created by keeping each image’s position the same, but changing the sequence within a triplet. For example, a foil could be made up of image sequence GBF, where each image within a triplet appears in the same position as they did before, but the sequence is new. The triplet and foil sequences were presented on each trial in the same manner as during familiarization, separated by a 1250 ms pause. The order of presentation was randomly chosen with equal likelihood. Observers pressed “A” if the first sequence seemed more familiar and “X” if the second sequence seemed more familiar.

### Results

#### Test Accuracy (Offline Measure of Learning)

**FIGURE 4:**
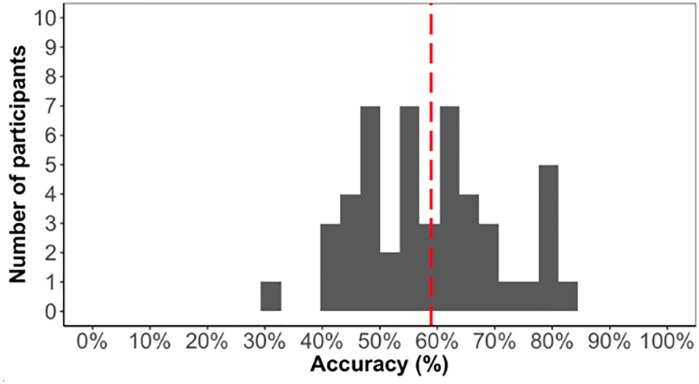
Distribution of test phase accuracy in Experiment 1.

As we are interested in individual differences, we first confirmed that the task elicited learning otherwise individual differences in the task would not be meaningful. The average accuracy score on the test phase was low (59%) but significantly above chance [*t*(48) = 5.1, *p* < 0.001]. Above chance learning overall suggested that participants learned the triplet sequence (Figure 4).

#### Pupil Change Across Triplet Presentation (Online Measure of Learning)

Having confirmed overall learning in the task and a wide-range of accuracy scores (i.e., our offline measures) during test phase, we went on to analyze pupil diameter obtained during familiarization (i.e., our online measure). We sought to determine whether the magnitude of the pupil change varies according to participant accuracy, image position (i.e., if it’s the first, second, or third image in a triplet), and number of times seen, and participant as a random effect. We ran two generalized linear mixed models (GLMMs), one using the average pupil change during the anticipatory period and the other using the average pupil change during the viewing period as the dependent variable for a single trial. We found that accuracy was not a significant predictor of pupil change for either anticipatory (β = −1.3e-2, *p* = 0.29) or viewing period (β = −2e-2, *p* = 0.7). To confirm whether the data favors the null hypothesis, we conducted a Bayes factor analysis comparing the GLMM with and without accuracy as a predictor. We found an extremely small Bayes factor of 0.02 for the anticipatory period and 0.05 for the viewing period, which suggests the data strongly favors the model without accuracy as a predictor. Overall, this lack of relationship suggest that participants’ pupil change during learning is not related to offline learning performance during the test phase.

**FIGURE 5:**
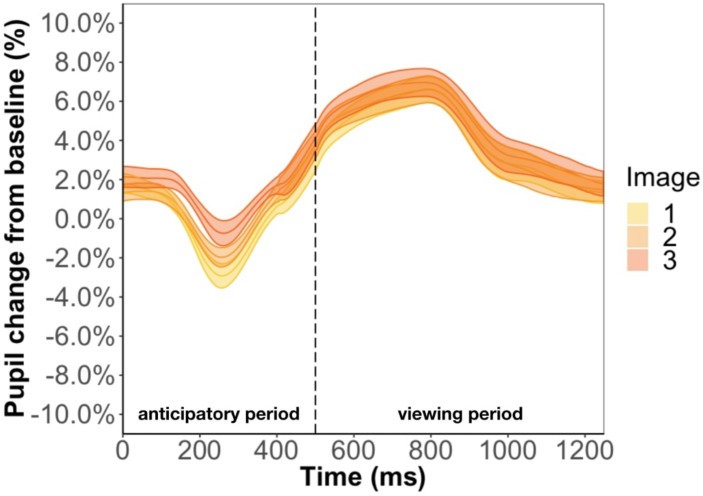
Pupil timecourse during viewing of a triplet in Experiment 1. The anticipatory period lasted for 500 ms. The dashed vertical line indicates when the image appears on the screen. The viewing period lasted for 750 ms, for a total trial duration of 1250 ms. The images in a triplet are overlaid on top of each other to visualize the pupil change within a triplet.

Focusing on the image position within the triplet, we found that image position was a significant predictor of pupil change during the anticipatory period (β = 6.3e-3, *p* < 0.001) but not the viewing period (β = 2.5e-3, *p* = 0.13) (Figure 5). The more predictable an image, the smaller the pupil constriction during the anticipatory period. Specifically, the anticipatory period appeared to reflect anticipation of the upcoming image because the ordering of pupil constriction from smallest to largest was image one, two, three. Furthermore, we found a familiarity effect on pupil diameter such that the number of times you see the same stimulus significantly increases pupil change for both the anticipatory (β = 4e-4, *p* < 0.05) and viewing period (β = 1.1e-3, *p* < 0.001).

#### Pupil Change Using Each Image as Their Own Baseline (Online Measure of Learning)

The analyses in the previous subsection found that pupil responses during the anticipatory period vary with triplet position. The data used for this finding employed a single baseline period per triplet (the anticipatory period for the first image). In this subsection, we reanalyze this data with each image’s anticipatory period as their own baseline and find that this effect does not persist.

We ran two regression models, one using the average pupil change during the anticipatory period and the other using the average pupil change during the viewing period as the dependent variable. For the anticipatory period, no predictor was significant (image position: β = −1.7e-5, *p* = 0.27; accuracy: β = 3.1e-4, *p* = 0.63; familiarity: β = −1.2e-6, *p* = 0.52). For the viewing period, we found a familiarity effect (β = 7.3e-4, *p* < 0.001) but no effect of image position (β = 3.7e-4, *p* = 0.79) and accuracy (β = 9.6e-3, *p* = 0.85). These results demonstrate that a change in baseline selection can alter the results because the effect of position and familiarity during the anticipatory period is no longer significant. We will return to the question of proper baseline in later experiments.

#### Siegelman et al. (2018) Online Measure of SL

We found that the trajectory of the online measure for Experiment 1 was significant for the anticipatory period (*r* = −0.41, *df* = 22, *p* = 0.046) and approaching significance for the viewing period (*r* = −0.38, *df* = 22, *p* = 0.06). However, results revealed a non-significant relationship between Siegelman et al. (2018)’s online measure of SL and accuracy on test phase (anticipatory period: *r* = 0.16, *df* = 47, *p* = 0.26, viewing period: *r* = 0.025, *df* = 47, *p* = 0.87). These results suggest that pupil change is not sensitive enough to reflect learning mechanisms in VSL.

#### Exploratory Analyses Suggested by Reviewers

We conducted exploratory analyses, as suggested by our Reviewers, to rule out alternative explanations. First, slower, tonic changes in pupil size were also investigated by investigating the unbaselined pupil size using the same GLMMs (Supplementary S1. Experiment 1 and S2. Experiment 1). We find the same pattern of effects as using the baselined pupil response which rules out the possibility that tonic pupil diameter is the better measurement of online learning. Second, a separate analysis using trials from the second half of the familiarization was conducted (Supplementary S3. Experiment 1 and S4. Experiment 1), to confirm that the mixed findings were not a result of including early trials, where learning might not have occurred. These results also converged the results of the main analyses suggesting that it is not slow or weak learning that is reducing the signal in the pupil response. Lastly, to confirm that the inclusion of poor learners did not obfuscate the results, we conducted identical analyses using the group of participants that performed above median, which had a median accuracy of 74%. This group also resulted in similar findings (Supplementary S5. Experiment 1 and S6. Experiment 1).

### Discussion

In Experiment 1, we found that pupil change during VSL familiarization phase does not predict accuracy during VSL test phase. We also found that pupil change during the anticipatory period reflects image position in a triplet. Specifically, as participants view the least predictable image to the most predictable image, pupil constriction weakens. This change in pupil constriction during the anticipatory period was an unexpected result for two reasons. First, the period which we find this shift in pupil change was unexpected because previous research found pupil change to reflect prediction error after stimulus presentation. In the current study, we found it before stimulus presentation and confirmed that it is not a result of participants not paying attention to the stimulus (Supplementary Figure S7). Second, the direction of pupil change was opposite of what we hypothesized. Previous research has shown that pupil dilation reflects prediction error (Nassar et al., 2012; Zhang et al., 2018), such that the larger the prediction error the larger the pupil dilation. In this task, we found that pupil constriction might be related to prediction error.

We also found an influence of familiarity such that pupil change increased as the task progressed, which contradicts well-established findings that larger pupil dilation is a signal for surprise (Preuschoff et al., 2011; Nassar et al., 2012; Kloosterman et al., 2015). This unexpected finding might be caused by changes in tonic pupil diameter (i.e., baseline). Specifically, studies have shown a strong negative correlation between tonic pupil diameter and phasic pupil change (e.g., Gilzenrat et al., 2010; Murphy et al., 2011), which is also confirmed in our data set (*r* = −0.33, *df* = 13504, *p* < 0.001). Therefore, because tonic pupil size is larger during the early trials of the experiment and decreases with time (*r* = −0.63, *df* = 283, *p* < 0.001), it likely explains why pupil change is small but increases as the task progressed. Overall, the results of Experiment 1 are mixed: a promising finding with respect to the pupil differences during the anticipatory period but it does not appear to be related to individual differences in performance at test. We move on to Experiment 2 to attempt to replicate the promising results to confirm whether they are veridical measures of VSL.

## Experiment 2: SL With Paired Images and Recognition Test

In Experiment 1, the difference in pupil change occurred in an unexpected direction and location. We found pupil change increased with predictability and occurred during the anticipatory period before a stimulus (i.e., in the ISI following the previous stimulus). Moreover, pupil change during familiarization was not correlated with accuracy during test. For these reasons, we sought to replicate and extend these findings. We conducted another VSL study using paired and unpaired images, instead of triplets, and a yes/no recognition test during test phase instead of 2AFC discrimination test. Having both paired and unpaired images allowed us to look at how the pupil change for a predicting image, predicted image and a completely irrelevant image (i.e., unpaired). In particular, the unpaired image helps us disentangle pupil changes that are caused solely by familiarity versus changes in predictability arising from SL. Furthermore, we used a familiarity test with the intention of making the test phase easier to increase the likelihood of learning and performance during the test phase. Again, Siegelman et al. (2018) online measure of SL was also calculated for both the anticipatory and viewing period.

### Procedure

#### Familiarization Phase

Participants watched a sequence of 288 images. The sequence was composed of pairs, or sequences of two images that always appeared in the same order, interleaved with unpaired (i.e., random) images. We randomly assigned the 12 fractal images into 4 pairs (AB, CD, EF, GH) and the remaining 4 images as the unpaired images (I,J,K,L), for every subject. The full sequence was generated by randomly interleaving eight repetitions of each paired and unpaired image with the constraint that no image could be repeated sequentially (e.g., AB-AB or AB-I-I).

#### Test Phase

Participants completed 32 untimed test trials. Half of the test trials were foils and the other half were actual pairs. The foil pairs were created by keeping each image’s position the same, but changing the sequence within a pair. For example, a foil could be made up of image sequence AF, where each image within a pair appears in the same position as they did before, but the sequence is new. Observers pressed “A” if the sequence was old and “X” if the sequence was new.

### Results

#### Test Accuracy (Offline Measure of Learning)

**FIGURE 6:**
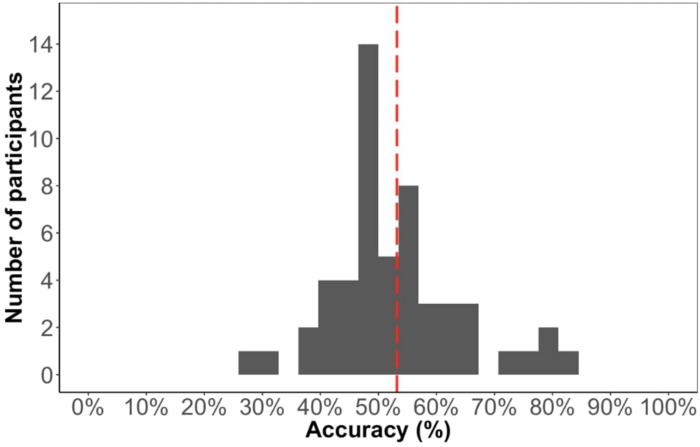
Distribution of test phase accuracy in Experiment 2.

The average accuracy score on the test phase was 53% (Figure 6), which is significantly above chance [*t*(52) = 2.05, *p* < 0.05], suggesting that participants learned the paired sequence during familiarization. However, the performance on this test is significantly lower compared to Experiment 1 [*t*(99) = 2.5, *p* < 0.05], suggesting that the change in familiarization from triplets to pairs and/or the change in test format made it more difficult to learn.

#### Pupil Change Across Triplet Presentation (Online Measure of Learning)

The purpose of Experiment 2 was to replicate and extend the findings from Experiment 1.

There are two principled ways to look at the data for this experiment with the goal of convergent findings. The two methods differ based on the trials included and how we baseline the pupil size. If pupil change is a valid online measure of SL, we should find the same effects across both methods.

For method one, we analyzed only the patterned sequence (i.e., paired images) and disregarded the unpaired images. Furthermore, we used the anticipatory period for the first image in a pair as baseline, which keeps the baseline selection consistent with Experiment 1. We ran two regression models: one using the average pupil change during the anticipatory period and the other using the average pupil change during the viewing period as the dependent variable. For both models, we included participant accuracy, image position (i.e., if it’s the first or second image in a pair), and number of times seen, as the independent variables and participant as a random effect.

**FIGURE 7:**
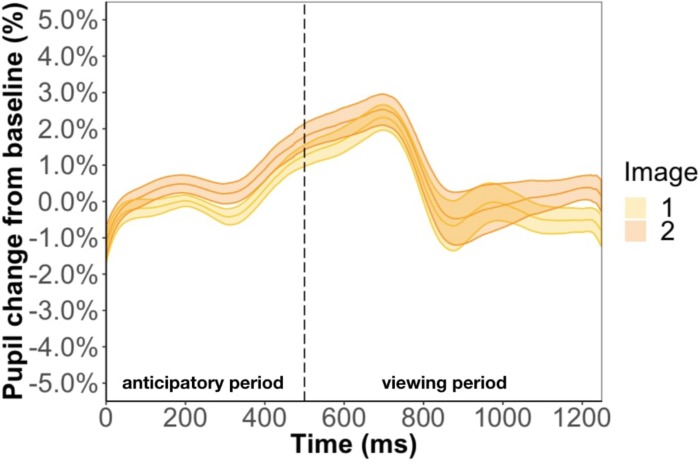
Pupil timecourse in Experiment 2 using the anticipatory period of the first image in a pair. The unpaired image was removed from analysis.

Replicating the result of Experiment 1, we found that pupil change during the anticipatory (β = −9.7e-3, *p* = 0.35) and viewing period (β = −5.3e-3, *p* = 0.85) was not related to test phase accuracy. We also conducted a Bayes factor analysis comparing the GLMM with and without accuracy as a predictor. We found an extremely small Bayes factor of 0.01 for the anticipatory period and 0.03 for the viewing period, which suggests the data strongly favors the model without accuracy as a predictor. Inconsistent with the results of Experiment 1, we found image position to be a significant predictor of pupil change during the anticipatory period (β = 4.9e-3, *p* < 0.01) and viewing period (β = 7.4e-3, *p* < 0.01) (Figure 7). In other words, the predicted image had a larger pupil change than the predicting image for the entire trial instead of just the anticipatory period, which is what we found in Experiment 1. Furthermore, we found a familiarity effect on pupil change during the viewing period (β = 3.4e-4, *p* < 0.05), but not anticipatory period (β = 2.4e-5, *p* = 0.80), whereas in Experiment 1 we found a familiarity effect for both periods.

**FIGURE 8:**
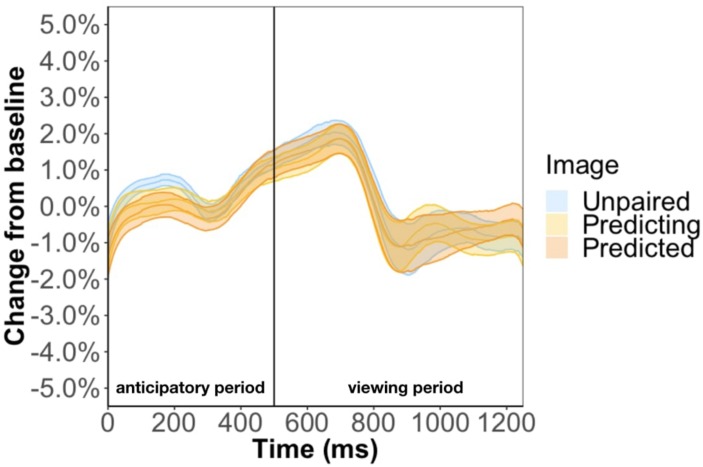
Pupil timecourse in Experiment 2 using the anticipatory period of the most recent unpaired image as basline.

Method two was a more rigorous analysis and involved keeping the unpaired image so that we could disentangle the effect of familiarity from prediction. For this method, the baseline was the anticipatory period of the most recent unpaired image. However, this baseline selection led to undistinguishable pupil changes between the three types of images (Figure 8). Specifically, the anticipatory pupil difference we found in Experiment 1 goes away suggesting that this effect is not a result of predictability *per se* but baseline selection. We tackle this issue more directly in Experiment 3.

#### Pupil Change Using Each Image as Their Own Baseline (Online Measure of Learning)

We consider method one results using a different baseline selection where each image’s anticipatory period is their own baseline. We did not conduct this analysis for method two because of the lack of significant results. For the anticipatory period, no predictor was significant (image position: β = 5e-6, *p* = 0.52; accuracy: β = 1e-5, *p* = 0.88; familiarity: β = 1.6e-7, *p* = 0.66). For the viewing period, we found a familiarity effect (β = 3.6e-4, *p* < 0.01) and image position (β = 7.1e-3, *p* < 0.01) but no effect of accuracy (β = 2.2e-3, *p* = 0.93). Again, altering the baseline period drastically altered the results.

#### Siegelman et al. (2018) Online Measure of SL

We calculated Siegelman et al. (2018) online measure of SL using just the paired images. We found that the trajectory of the online measure was not significant for both the anticipatory (*r* = 0.006, *df* = 34, *p* = 0.97) and viewing period (*r* = −0.16, *df* = 34, *p* = 0.35). Furthermore, results revealed a non-significant relationship between online measure of SL and accuracy on test phase (anticipatory period: *r* = 0.14, *df* = 50, *p* = 0.31, viewing period: *r* = 0.03, *df* = 50, *p* = 0.84). These results provide further evidence that pupil change is not reflecting the learning mechanisms supporting VSL.

### Discussion

In Experiment 1, the difference in pupil change occurred in an unexpected direction and location. We found pupil change increased with predictability and occurred during the anticipatory period before a stimulus (i.e., in the ISI following the previous stimulus). Moreover, pupil change during familiarization was not correlated with accuracy during test. For these reasons, we sought to replicate and extend these findings.

In Experiment 2 we conducted another VSL study using paired and unpaired images, instead of triplets, and a yes/no recognition test during test phase instead of 2AFC discrimination test.

The changes in experimental procedure led to one consistent result and several inconsistent results. Using the first image of the paired images as baseline and removing the unpaired image, we replicated the main finding from Experiment 1: we found that pupil change during the anticipatory period reflected image position in a pair with a greater pupil change during more predictable pictures. Furthermore, the differentiable pupil changes across position, did not predict performance during test. Focusing on the inconsistent results, first, while we find an effect of position in the anticipatory period, we also found this effect during the viewing period which was not present in Experiment 1. Second, we found the familiarity effect during the viewing period but not anticipatory period. The last and most concerning result was when we used the anticipatory period of the most recent unpaired image as baseline, the anticipatory pupil difference we found in Experiment 1 disappeared. This inconsistent pattern of results led us to be skeptical that the pupil differences we found during the anticipatory period in Experiment 1 and Experiment 2 is a valid online measure of SL. Therefore, we conducted Experiment 3 to investigate whether pupil changes in the anticipatory period is a result of prediction or baselining.

## Experiment 3: VSL Using Triplet and Random Blocks

In Experiment 3, we investigated whether pupil changes in the anticipatory period are reflective of online learning processes or are an artifact of our baselining procedure. In other words, is the pupil change during anticipatory period related to participants’ learning (e.g., prediction for an upcoming image)? Or is it a result of simply choosing baseline to be the anticipatory period of the first image in a triplet or pair? To distinguish between the two alternatives, we used a within subject design to measure pupil change during a triplet block and a random block. Our hypothesis is if the differences in pupil change is due to stimulus prediction, then we should only see pupil difference in the anticipatory period during the triplet block. However, if the differences in pupil change is due to baselining then we should see the difference in both triplet and random block. Siegelman et al. (2018) online measure of SL was also calculated for both blocks.

### Methods

#### Procedure

The experiment was made up of two blocks: triplet and random. All participants first completed the triplet block, followed by the random block. We did not counterbalance the blocks, because successively presented sets of stimuli can bias or reduce learning (Gebhart et al., 2009). Participants watched a sequence of 480 fractal images (240 images per block).

In the triplet block, the sequence followed the same rules as the familiarization phase of Experiment 1. In the random block, the full image sequence was presented in a complete randomized order, with the exception that no image could be repeated sequentially. We did not include a test phase for Experiment 3.

### Results

#### Pupil Change Across Triplet Presentation (Online Measure of Learning)

**FIGURE 9:**
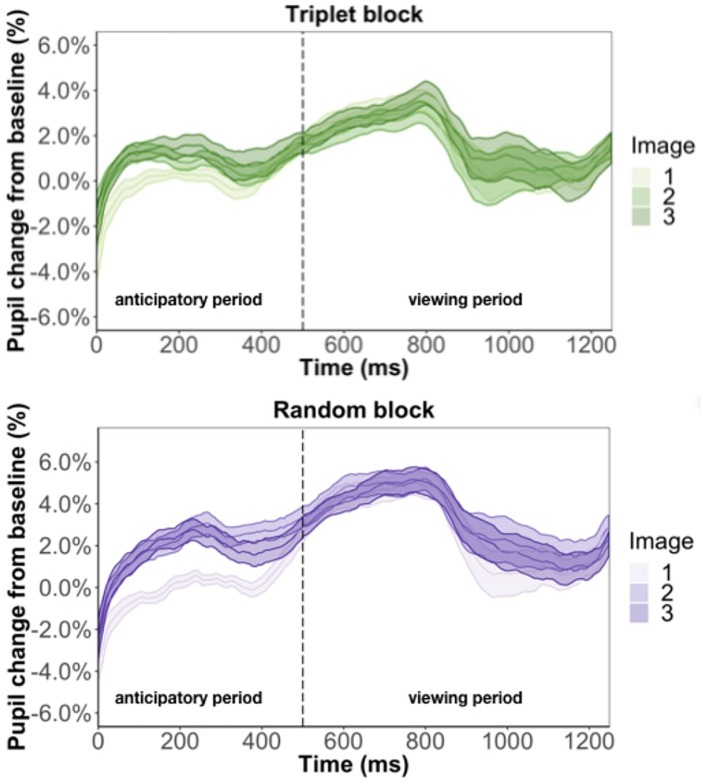
Pupil time-course during viewing of a triplet in Experiment 3. The anticipatory period lasted for 500 ms. The dashed vertical line indicates when the image appears on the screen. The viewing period lasted for 750 ms, for a total trial duration of 1250 ms. The images in a triplet are overlaid on top of each other to visualize the pupil change within a sequence of three images.

We ran two regression models to look at what predicts pupil change during the anticipatory period. One model used average pupil change during the anticipatory period of the triplet block and the other model used the average pupil change during the anticipatory period of the random block (Figure 9). If the pupil change in anticipatory period in Experiment 1 is due to stimulus prediction, we expect to find image position as a significant predictor for the triplet block but not the random block. If the pupil change in anticipatory period is due to temporal prediction, we expect to find image position as a significant predictor for both the triplet and random block. To test what was the cause of the pupil change, we regressed average pupil change on image position, and number of times seen for both models. In terms of image position, because there is no picture predictability in the random block we simply grouped three consecutive images together to form a “random triplet,” whereas the group of three images in the triplet block was a “patterned triplet.”

We found that image position was a significant predictor of pupil change during the anticipatory period for both the triplet block (β = 5.3e-3, *p* < 0.001) and random block (β = 8.6e-3, *p* < 0.001). In other words, pupil dilation was smallest for the first image in a triplet and largest for the third image in a triplet, with the second image falling in the middle, for both the triplet block and random block. This suggests that the pupil difference during the anticipatory period is likely an effect of where we chose to baseline, instead of stimulus prediction, because we find this difference in the random block, where the stimuli are not predictable. Furthermore, we found a familiarity effect on pupil change during the anticipatory period for the triplet block (β = 6e-4, *p* < 0.01) but not random block (β = 7.2e-5, *p* = 0.79). This suggests that the unstructured nature of the random block caused participants to not recognize the individual stimuli and view each appearance as it was their first time seeing the image.

We also ran two regression models to look at what predicts pupil change during the viewing period. Replicating Experiment 1, we found evidence for a familiarity effect on pupil change during the triplet block (β = 7.5e-4, *p* < 0.01) and we did not find effect of image position during the viewing period (β = 6.1e-4, *p* = 0.71). This result suggests that the inconsistent findings from Experiment 2 is caused by changing the triplets to pairs.

#### Pupil Change Using Each Image as Their Own Baseline (Online Measure of Learning)

Using each image’s own anticipatory period as baseline, we found that during the anticipatory period, image position was almost a significant predictor (β = −1.86e-5, *p* = 0.088) but no familiarity effect (β = −1.5e-6, *p* = 0.33). However, for the random block, image position was also almost a significant predictor (β = −2.5e-5, *p* = 0.07) and there was a familiarity effect (β = −4.6e-6, *p* = 0.02). During the viewing period, there was no significant predictors for either blocks.

#### Siegelman et al. (2018) Online Measure of SL

We found that the trajectory of the online measure was not significant for the triplet block (anticipatory period: *r* = −0.40, *df* = 18, *p* = 0.08, viewing period: *r* = −0.23, *df* = 17, *p* = 0.34) or random block (anticipatory period: *r* = 0.03, *df* = 18, *p* = 0.9, viewing period: *r* = 0.14, *df* = 18, *p* = 0.55). Importantly, when comparing the learning trajectories between the triplet and random block, we found no significant interaction. This lack of difference suggests that participants did not differ in how well they learned the statistical structure for an input with structure versus an input without structure.

### Discussion

In Experiment 3, we found pupil change during the anticipatory period reflects image position for both the triplet and random block. This result suggests that the pupil change during the anticipatory period in both Experiment 1 and Experiment 2 is a result of baselining rather than stimulus predictability. Based on the results of Experiment 3, we can reinterpret the pupil difference between image position during the anticipatory period of Experiments 1 and 2 as purely due to baseline selection, not predictability.

## General Discussion

In three experiments, we measured participants’ pupil diameter using VSL. In Experiment 1, we employed a VSL task using images grouped into triplets during familiarization and 2AFC task during test. In Experiment 2, we employed a VSL task using paired and unpaired images during familiarization and a familiarity task during test phase. In Experiment 3, we employed a VSL task without the test phase and showed participants a sequence of images first composed of triplets (i.e., triplet block) followed by a sequence of random images (i.e., random block). Overall, we found that pupil diameter is not a good online measure to use during SL given that it does not predict offline measures of learning (i.e., performance during test phase) and varies substantially according to baseline selection (for summary of results see Table 1). Finally, drawing from previous pupillometry studies looking at prediction and prediction error, we propose that these negative effects arise from the fact that the pupil response unfolds too slowly to be compatible with the fast nature of VSL.

**TABLE 1:**
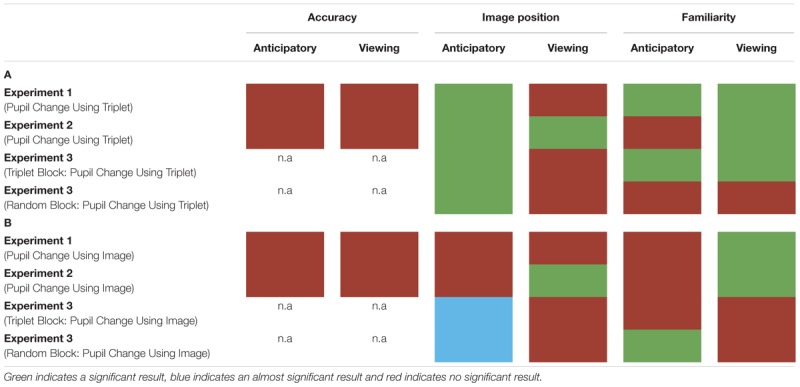
Overview of the two different baseline selection results for all experiments.

Red indicates the result was not significant, green indicates the result was significant, and blue suggests approaching significance. (A) Pupil change was calculated for each triplet by selecting a single baseline period (the first image’s anticipatory period) per triplet. (B) Pupil change was calculated by using each image’s anticipatory period as their own baseline.

First, if pupillometry was a good measure of prediction and learning during VSL we would expect it to be sensitive enough to distinguish the participants that learned the patterned sequence from those that didn’t. However, in Experiment 1 and Experiment 2, we found that pupil change during familiarization (i.e., learning) did not predict performance during test phase. One possibility for this is that the pupil response does not reflect the relevant online learning mechanisms. We argue that this is not likely to be the case: as reviewed above, pupillometry has been shown to be sensitive to processes that are part of prediction such as prediction error (Nassar et al., 2012; Koenig et al., 2018; Zhang et al., 2018) and updating (O’Reilly et al., 2013). Thus, it is likely that the pupil response is reflecting relevant online processes during learning.

Second, pupil diameter is not suitable for SL because where we chose to set baseline drastically altered pupil change. In Experiment 1, we found that pupil change during the anticipatory period reflected image position in a triplet such that the pupil constriction was smallest for the third image (i.e., the most predictable image) in a triplet and largest for the first image (i.e., the least predictable image) in a triplet. In Experiment 2, we used paired and unpaired images instead of triplet images because we wanted to disentangle pupil change associated with familiarity from prediction. However, data analysis using the anticipatory period of the unpaired image as baseline led to undistinguishable pupil responses. On the other hand, when we removed the unpaired image from data analysis and used the anticipatory period of the first image in a pair as baseline, we found the same pupil difference in the anticipatory period as Experiment 1. To determine whether this pupil difference during the anticipatory period was a result of stimulus prediction or baseline selection we conducted Experiment 3. The results of Experiment 3 revealed a significant difference in pupil change during the anticipatory period in both the triplet block and random block (i.e., no predictive processing) suggesting the underlying cause for this shift baseline selection rather than stimulus prediction.

**FIGURE 10:**
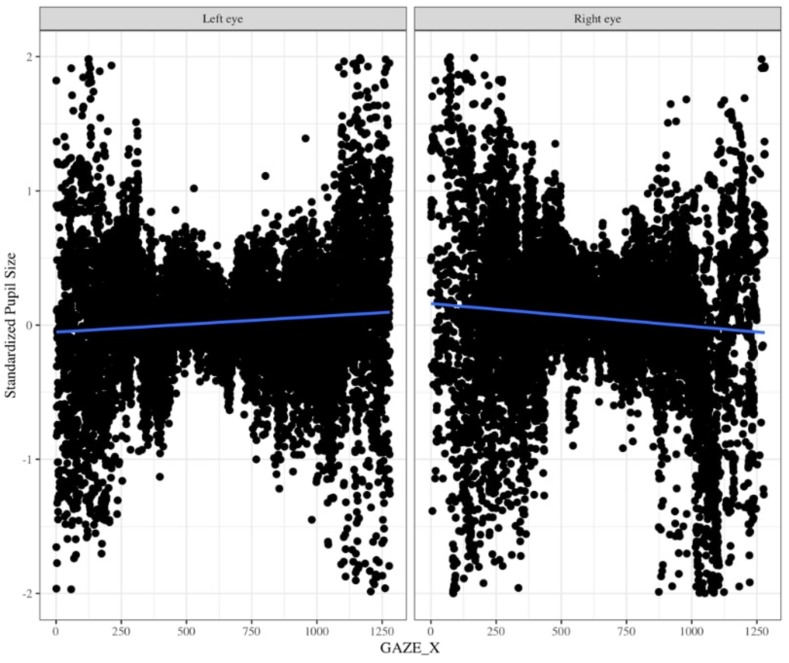
Changes in pupil diameter based on X coordinate eye position for left eye **(left panel)** and right eye **(right panel)**. Pupil diameter was z-scored to the infant’s average pupil diameter for the entire experiment.

One might suggest that there is no need to baseline pupil diameter; however, there are two reasons why it is very important to baseline pupil size. The Eyelink 1000 eyetracker records the pupil diameter in arbitrary units in the range of 400 to 16,000 units to represent pupil diameter with large differences across individuals and within individuals throughout the experiment. Without baselines, this substantial variation will likely wash out any systematic differences in pupil size induced by your experimental task. Moreover, this arbitrary unit can be tricky to convert to mm because the coefficient of proportionality depends on the experimental layout (i.e., the relative positions of the camera, eye, and monitor) (Hayes and Petrov, 2015). Using an artificial eye, and selecting “Left” on the Eyelink 1000 eyetracker, Hayes and Petrov (2015) found that pupil diameter recordings tend to increase as gaze position moves from left to right throughout the middle of the screen. Using data, we collected for a separate infant study in which we specifically counterbalanced which eye we collected data from, we found the same pattern of pupil deviation for the left eye, but for pupil diameter that was recorded using the “Right” eye we found the opposite trend (personal communication Gabriel Xiao, Figure 10). Thus, pupil size varies for a given x coordinate that the eye is pointed to and moreover, this relationship changes based on whether one is recording from the right vs. left eyes. Therefore, it is important to calculate percent change from baseline.

In addition, depending on the type of analysis, baselining is important because the pupil diameter tends to increase throughout the experiment due to drift. It is important to note that even though we baselined our pupil diameter for all our studies, we still found increased pupil change throughout the experiment. Results from our studies demonstrate the influence of familiarity on pupil change. For all three experiments, we found results supporting research suggesting that demonstrates larger pupil dilation for more familiar stimuli (Kafkas and Montaldi, 2012, 2015).

But this increase in pupil change could have been even larger if we didn’t baseline. Therefore, baselining can help reduce the effect of drift on pupil diameter.

However, deciding which portion of the task to use for baseline is difficult. Generally, which portion of the task you choose to be your baseline depends on the task. Some studies have used the beginning of the experiment (Tsukahara et al., 2016), some studies use beginning of every trial or before stimulus onset (Murphy et al., 2016; Wetzel et al., 2016; Byers-heinlein et al., 2017; Koenig et al., 2018) some studies use the average pupil diameter for the entire experiment as baseline (Geng et al., 2015) and some compute z-score pupil size for the entire experiment (Kang et al., 2014; Kang and Wheatley, 2015). Ideally, one should baseline for each stimulus to avoid creating artifacts based on base-lining but this isn’t likely to happen for VSL because of the type of effects researchers are interested in. In other words, baselining pupil diameter for each trial can remove experimental effects.

Beyond the issues of baselining, we propose that there is a more fundamental issue: pupillometry may not be compatible with VSL due to the slow nature of the pupil change and the fast nature of SL tasks. Many studies that have revealed that the pupil response reflects processes like prediction error find these effects persisting many seconds after stimulus presentation. Indeed, studies that have included a long enough ISI for the pupil change to return to baseline (Lavín et al., 2014; Zhang et al., 2018) have been successful in using pupillometry in their respective task. However, all VSL tasks, however, present each stimulus for only 1000 ms or less, with an even shorter ISI, resulting in an extremely short trial (e.g., Kirkham et al., 2002; Turk-Browne et al., 2005; Abla and Okanoya, 2009) that prevents pupil change from fully returning to baseline. Longer ISIs in VSL tasks may substantially change the mechanisms supporting VSL and/or result in no learning. Therefore, we propose that the timing required by VSL studies (and SL studies more broadly) is not readily able to capture the aspects of the pupil response that reflect the relevant online processes.

This timing issue is reminiscent of the timing constraints of fMRI. fMRI has been used to investigate the mechanisms supporting VSL (e.g., Turk-Browne et al., 2010a) and has incorporated the longer timing necessary (e.g., 3–6 s ISIs). Using this design of fMRI style design may be a future avenue for merging pupillometry and VSL. However, given the substantial changes in these paradigms compared to standard VSL tasks and the difficulty in investigating VSL in developmental and differently-abled populations with these paradigms (i.e., because of the slow rate of stimuli and need for longer exposures), it raises the question of how useful pupillometry is as an online measure of VSL.

While pupillometry has been a highly fruitful online measure during a variety of tasks, we propose that it is severely limited in its application to investigating SL. Moving forward, researchers interested in the online mechanisms of SL can continue to experiment with the available methods. For adults, certainly fMRI is a fruitful alternative and the need for slower timing is complemented by the acquisition of a signal reflecting a myriad of systems in the brain. A review by Karuza et al. (2014a) argues that fMRI is an excellent online measure of learning as it can measure fluctuations in neural activity as learning unfolds over time, thereby allowing researchers to tap into the process through which internal representations undergo changes. Behaviorally, we have two RT measures available: click detection and self-paced paradigm. Comparing the two available alternatives, the self-paced paradigm seems to be the better option because there are less possible confounds given there isn’t a cover task. Overall, current neuroimaging and behavioral methods still have the ability to expand our current knowledge of SL and reveal the online mechanisms that occur during learning in SL and we propose they are superior to the use of pupillometry for VSL.

## Data Availability

The datasets generated for this study are available on request to the corresponding author.

## Ethics Statement

This study was carried out in accordance with the recommendations of the Institutional Review Board for Human Subjects at Princeton University and with written informed consent from all subjects. All subjects gave written informed consent in accordance with the Declaration of Helsinki. The protocol was approved by the Institutional Review Board for Human Subjects at Princeton University.

## Author Contributions

FZ and LE designed the study. FZ collected and analyzed data. FZ wrote the manuscript with input from LE.

## Conflict of Interest Statement

The authors declare that the research was conducted in the absence of any commercial or financial relationships that could be construed as a potential conflict of interest.
