# Supplementary material for: Opposing Timing Constraints Severely Limit the Use of Pupillometry to Investigate Visual Statistical Learning
Source: Front Psychol. 2019 Aug 6;10:1792. doi: 10.3389/fpsyg.2019.01792 (PMC6691770; doi:10.3389/fpsyg.2019.01792)

Supplementary Information

S1. Experiment 1: Pupil Change Across Triplet Presentation (Online Measure of Learning) using Unbaselined Pupil Diameter

We ran the same GLMMs for Experiment 1, using unbaselined pupil diameter, for the anticipatory and viewing period. For the anticipatory period, we found a familiarity effect ($\beta = -7.8$, $p < 0.001$) but no effect of image position ($\beta = -1.2$, $p = 0.81$) and accuracy ($\beta = -552.7$, $p = 0.58$). For the viewing period, we found a familiarity effect ($\beta = -7.2$, $p < 0.001$) but no effect of image position ($\beta = 1.2$, $p = 0.79$) and accuracy ($\beta = -555.5$, $p = 0.58$). Overall, we find that unbaselined pupil size during the entire trial decreased as a result of familiarity, which could be reflective of diminishing uncertainty. However, this decrease in unbaselined pupil size is not predictive of accuracy, so it is unlikely that this change is related to learning effects.

S2. Experiment 1: Siegelman et al. (2018) Online Measure of SL using Unbaselined Pupil Diameter

When we calculate Siegelman et al. (2018) measure of SL using unbaselined pupil diameter, we find that the trajectory of the online measure was not significant for Experiment 1 for both the anticipatory ($r = -0.15$, $df = 22$, $p = 0.48$) and viewing period ($r = -0.34$, $df = 22$, $p = 0.1$). Furthermore, results revealed a non-significant relationship between Siegelman et al. (2018)'s online measure of SL and accuracy on test phase (anticipatory period: $r = 0.21$, $df = 47$, $p = 0.14$, viewing period: $r = -0.015$, $df = 47$, $p = 0.92$). These results also indicate that unbaselined pupil diameter is also not an online measure for learning in VSL.

S3. Experiment 1: Pupil Change Across Triplet Presentation (Online Measure of Learning) using Second Half of Familiarization Trials

We ran the same GLMMs for Experiment 1, using the second half of trials, for the anticipatory and viewing period. For the anticipatory period, we found that the simplest model, with only image position as a predictor result in the best GLMM model. The model revealed that image position ($\beta = 9.5e\text{-}3$, $p < 0.001$) had a significant effect on pupil change. For the viewing period, we found a significant familiarity effect ($\beta = 1.4e\text{-}3$, $p < 0.05$), marginal image position effect ($\beta = 4.3e\text{-}3$, $p = 0.1$) but no effect of accuracy ($\beta = 4.2e\text{-}3$, $p = 0.94$).

S4. Experiment 1: Siegelman et al. (2018) Online Measure of SL using Second Half of Familiarization Trials

We calculated Siegelman et al. (2018) measure of SL using the second half of trials. Results showed the trajectory of the online measure was not significant for Experiment 1 for both the anticipatory ($r = -0.19$, $p = 0.56$) and viewing period ($r = 0.09$, $p = 0.52$). Furthermore, results revealed a non-significant relationship between Siegelman et al. (2018)'s online measure of SL and accuracy on test phase (anticipatory period: $r = 0.16$, $p = 0.28$, viewing period: $r = -0.08$, $p = 0.58$). Overall, we find that by investigating the second half of the experiments, where we can be fairly confident participants have learned, accuracy is still not predictive of pupil change and pupil change is not reflective of online learning.

S5. Experiment 1: Pupil Change Across Triplet Presentation (Online Measure of Learning) using Participants that Performed Above Median Split

We reanalyzed the data from Experiment 1, by focusing on the participants who performed above median (60%). This group of participants has a median accuracy of 74%. We ran the same GLMMs as Experiment 1. For the anticipatory period, we found that the simplest model, with only image position as a predictor result in the best GLMM model. The model revealed that image position (β = 6.2e-3, p < 0.01) had a significant effect on pupil change. For the viewing period, we found a significant effect of familiarity (β = 1.3e-3, p < 0.001) but no effect of accuracy (β = -6.7e-3, p = 0.59) or image position (β = 2.4e-3, p = 0.33). These results confirm that it is not the inclusion of poor learners (as measured by post-test performance) that obfuscates the pupillometry effects.

S6. Experiment 1: Siegelman et al. (2018) Online Measure of SL using Participants that Performed Above Median Split

We also calculated Siegelman et al. (2018) measure of SL. Results showed the trajectory of the online measure was not significant for Experiment 1 for both the anticipatory (r = -0.22, p = 0.29) and viewing period (r = -0.24, p = 0.26). Furthermore, results revealed a non-significant relationship between Siegelman et al. (2018)'s online measure of SL and accuracy on test phase (anticipatory period: r = 0.36, p = 0.1, viewing period: r = 0.15, p = 0.51). Overall, we find that by investigating the good learners, with a median accuracy of 74%, pupil change is still not a sensitive measure of online learning.

Figure S7. Average gaze position for trial in Experiment 1. A. Plot shows where along the horizontal dimension of the screen participants were looking. The horizontal black line in both plots indicates the center of the screen, where the visual stimulus appeared. **B.** The second plot

shows where along the vertical dimension of the screen participants were looked. Taken together, the plots show that participants were looking at the center of the screen during the entire trial, confirming changes in pupil change is not a result of eye movement.

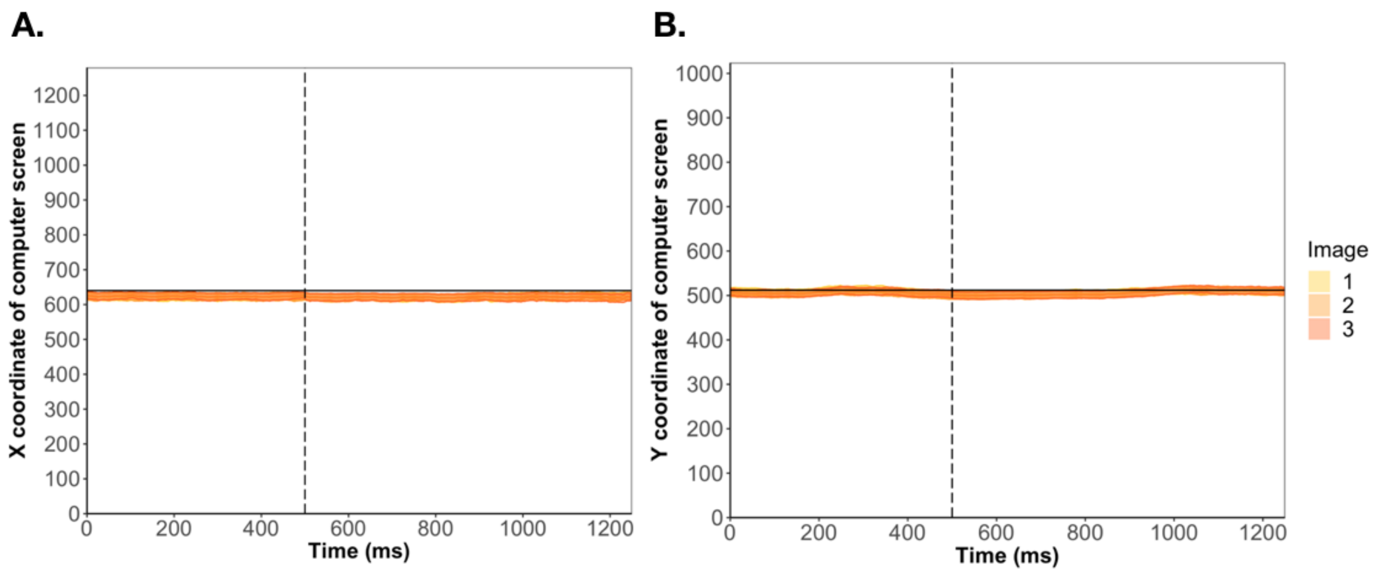

Supplement: Supplementary file 1 [file Data_Sheet_1.pdf]
